# Supplementary material for: Predicting Short-Term Survival after Gross Total or Near Total Resection in Glioblastomas by Machine Learning-Based Radiomic Analysis of Preoperative MRI
Source: Cancers (Basel). 2021 Oct 9;13(20):5047. doi: 10.3390/cancers13205047 (PMC8533879; doi:10.3390/cancers13205047)
Supplement: Supplementary file 1 [file cancers-13-05047-s001.zip › cancers-1383638-Supplementary Materials.pdf]

Supplementary Table S1. The specific parameters of structural multiparametric MRI			
	Dataset		
MRI Sequence	TCIA	Institution 1	Institution 2
**T1CE**	TR/TE, 4.9–3285 ms/2.1–20 ms; slice thickness, 1–5 mm; spacing slice, 0.6–7.5 mm	TR/TE, 7.98 ms/2.57 ms; FOV, 220 x 220 mm; matrix, 512 x 512; slice thickness, 1 mm	TR/TE, 6.82 ms/2.16 ms; FOV, 320 x 320 mm; matrix, 512 x 512; slice thickness, 1.6 mm
**T1WI**	TR/TE, 352–3379 ms/2.75–19 ms; slice thickness, 1–5 mm; spacing slice, 2–7.5 mm.	TR/TE, 5.98 ms/1.83 ms; FOV, 220 x 220 mm; matrix, 512 x 512; slice thickness, 1.6 mm	TR/TE, 680 ms/7.63 ms; FOV, 320 x 320 mm; matrix, 512 x 512; slice thickness, 5 mm
**T2WI**	TR/TE, 700–6370 ms/15–120 ms; slice thickness, 1.5–5 mm; spacing slice, 1.5–7.5 mm	TR/TE, 5220 ms/96.12 ms; FOV, 220 x 220 mm; matrix, 512 x 512; slice thickness, 5 mm.	TR/TE, 4322 ms/124.25 ms; FOV, 256 x 256 mm; matrix, 512 x 512; slice thickness, 4.4 mm
**FLAIR**	TR/TE, 6000–11,000 ms/34.6–155 ms; slice thickness, 2.5–5 mm; spacing slice, 2–7.5 mm. The matrix size of all the MRI sequences was either 256 × 256 or 512 × 512.	TR/TE, 11000 ms/142.43 ms; FOV, 220 x 220 mm; matrix, 512 x 512; slice thickness, 4 mm	FLAIR : TR/TE, 9350 ms/142.56 ms; FOV, 320 x 224 mm; matrix, 512 x 512; slice thickness, 4 mm

TCIA = The Cancer Image Archive, T1WI = T1-weighted image, T1CE = contrast-enhanced T1WI, T2WI= T2-weighted image, FLAIR = Fluid-attenuated inversion recovery, TR = repetition time, TE= echo time, FOV = field of view.

Supplementary Table S2. Radiomic features description	
**Feature Family**	**Specific Features**
**Intensity Features (First-Order Statistics)**	Minimum<br>Maximum<br>Mean<br>Standard Deviation<br>Variance<br>Skewness<br>Kurtosis
**Histogram -based**	Bin Frequency
**Volumetric**	Volume/Area
**Morphologic**	Elongation<br>Perimeter<br>Roundness<br>Eccentricity<br>Ellipse Diameter<br>Equivalent Spherical Radius
**Grey Level Co-occurrence Matrix (GLCM)**	Energy (Angular Second Moment)<br>Contrast (Inertia)<br>Joint Entropy<br>Homogeneity (Inverse Difference Moment)<br>Correlation<br>Variance<br>SumAverage<br>Auto<br>Correlation
**Grey Level Run-Length Matrix (GLRLM)**	SRE<br>LRE<br>GLN<br>RLN<br>LGRE<br>HGRE<br>SRLGE<br>SRHGE<br>LRLGE<br>LRHGE
**Neighborhood Grey-Tone Difference Matrix (NGTDM)**	Coarseness<br>Contrast<br>Busyness<br>Complexity<br>Strength
**Grey Level Size-Zone Matrix (GLSZM)**	SZE<br>LZE<br>GLN<br>ZSN<br>ZP<br>LGZE<br>HGZE<br>SZLGE<br>SZHGE<br>LZLGE

	LZHGE<br>GLV<br>ZLV
**Lattice -based**	Selected features<br>Feature Maps

Supplementary Table S3. Hyperparameters of machine learning classifiers used in this study.

Classifier	Hyperparmeters
**SVM**	Cost = 1.0; Regression loss epsilon = 0.10; Kernel = linear; Numerical tolerance = 0.0010; Iteration limit = 100
**kNN**	Number of neighbors = 5; Metric = Euclidean; Weight = uniform
**Random Forest**	Number of trees = 5; Replicable training = yes; Attributes at each split = 5; Limit depth of individual trees = 3.
**Logistic regression**	Regularization type = LASSO (L1)
**Naïve Bayes**	Type = Gaussian, Priors = None; Variable smoothing = 1e-9
**Neural Network**	Neurons in hidden layers = 100; Activation = ReLu; Solver = Adam; Regularization alpha = 0.001; Maximal number of iterations = 200; Replicable training = yes.

SVM = Support Vector Machine, kNN = k-Nearest Neighbor, LASSO = Least Absolute Shrinkage and Selection Operator.

Supplementary Table S4. Model performance grouped by feature selection filter and machine learning classifier on training dataset

Classifier	Filter	AUC	CA	Precision	F1
**Naive Bayes**	Information Gain	0.884	0.818	0.883	0.837
	Gini Index	0.886	0.825	0.877	0.842
	FCBF	0.885	0.804	0.879	0.826
**k-Nearest Neighbor**	Information Gain	0.925	0.902	0.902	0.886
	Gini Index	0.929	0.895	0.895	0.876
	FCBF	0.904	0.902	0.895	0.893
**Neural Network**	Information Gain	0.906	0.902	0.912	0.882
	Gini Index	0.911	0.909	0.918	0.892
	FCBF	0.894	0.902	0.897	0.890
**Random Forest**	Information Gain	0.978	0.944	0.948	0.939
	Gini Index	0.985	0.972	0.973	0.971
	FCBF	0.983	0.937	0.937	0.932
**Support Vector Machine**	Information Gain	0.802	0.853	0.828	0.803
	Gini Index	0.817	0.867	0.885	0.821
	FCBF	0.842	0.860	0.846	0.817
**Logistic Regression**	Information Gain	0.823	0.839	0.715	0.772
	Gini Index	0.823	0.846	0.797	0.788
	FCBF	0.853	0.839	0.715	0.772

AUC: area under the curve; CA: classification accuracy; FCBC: fast correlation based filter.

Supplementary Table S5. Kaplan-Meier analysis based on Random Forest Survival risk groups

Sample	OOB-CHF Cutpoint	Risk groups and number of cases	Median OS (IQR) days	Log-rank Test	
				χ^2	*p*
**Training datset**	0.6836856	Low = 86	466 (283)	19.9	< .001
		High = 57	331 (236)		
**Testing dataset**	0.6836856	Low = 43	486 (314)	7	.008
		High = 17	155 (236)		

OS= Overall survival. IQR= interquartile range, OOB-CHF = out of bag cumulative hazard function.

Supplementary Table S6. Methodologic quality evaluation of our study by the Radiomic Quality Score	
Scoring item	Points
Image protocol quality	1
Multiple segmentations	1
Phantom study on all scanners	0
Imaging at multiple time points	0
Feature reduction or adjustment for multiple testing	3
Multivariate analysis with nonradiomic features	1
Detect and discuss biologic correlates	0
Cutoff analysis	1
Discrimination statistics	2
Calibration statistics	1
Prospective study registered in trial data base	0
Validation	5
Comparison with criterion standard	0
Potential clinical utility	2
Cost-effectiveness analysis	0
Open science and data	2
**Total points**	**19/36 (53 %)**

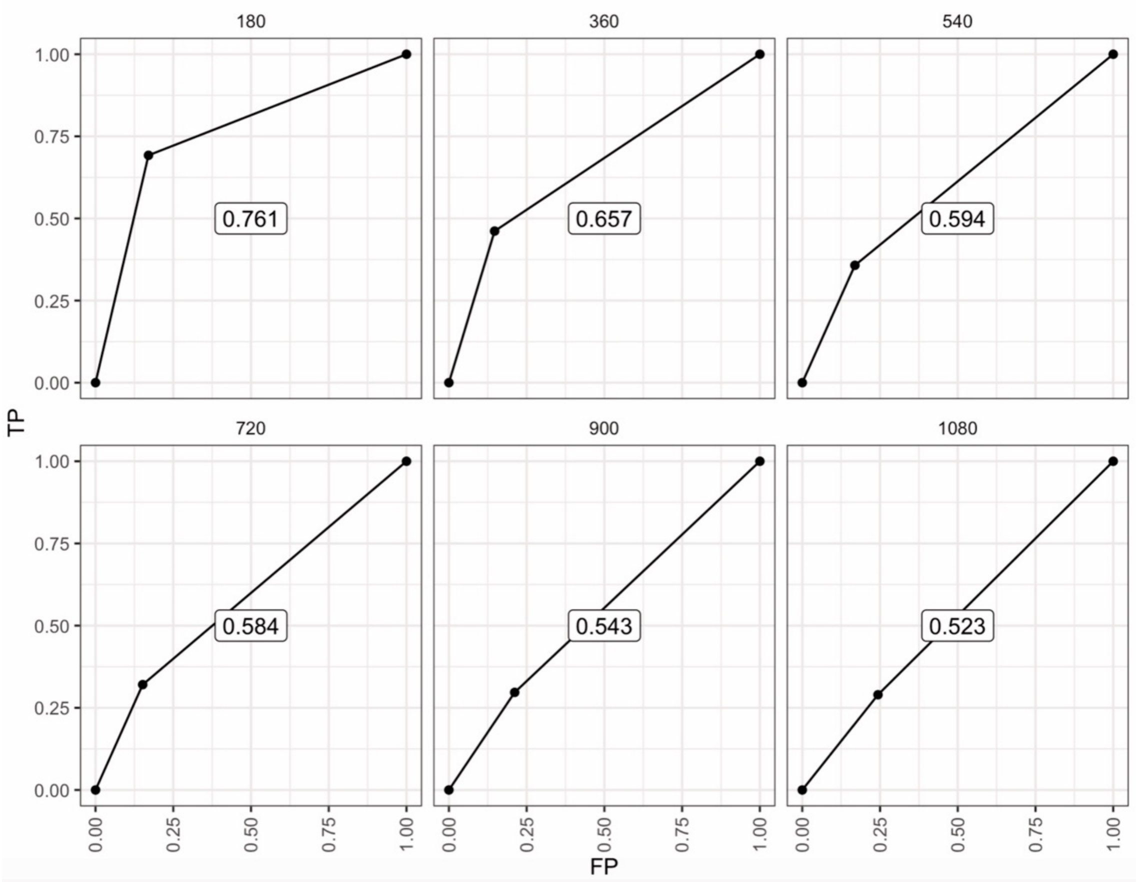

Supplementary Figure S1. Time-dependent receiver operating characteristics (ROC) curve of the Random Survival Forest survival model in the testing data set. Time in days appears at the top of each panel. TP = true positives, FP = False positives.
